# Editorial: “Ion channels and mental illness: exploring etiology and pathophysiology in major psychiatric disorders”

**DOI:** 10.3389/fgene.2015.00152

**Published:** 2015-04-24

**Authors:** Kathleen D. Askland

**Affiliations:** Psychiatry, Research and Academics, Waypoint Centre for Mental Health Care/University of TorontoPenetanguishene, ON, Canada

**Keywords:** ion channels, sequencing, etiology, pathophysiology, neuropsychiatry, major mental illness

This Research Topic, Ion Channels and Mental Illness: Exploring etiology and pathophysiology in major psychiatric disorders, presents three original research manuscripts and five literature reviews that advance our understanding of the role of ion channels in the etiology and pathogenesis of psychiatric disease.

Gilling et al. (2013) present their original findings of a novel de novo truncating translocation of KCNQ3 in one subject with autism and of a very rare nucleotide substitution (rs74582884) in KCNQ3 in three new subjects with autism. The substitution was previously reported in patients with forms of epilepsy. Heterologous expression of mutant channels in Xenopus laevis oocytes demonstrated reduced current through the Kv7.3/Kv7.5 channel complex relative to wild type. They suggest that functional impairment of the Kv7.3/Kv7.5 channel complex may confer susceptibility to several neuropsychiatric disorders.

Judy et al. (2013) examine three bipolar disorder (BP) genome-wide association studies (GWAS) datasets for evidence of two-way interactions between ANK3 SNPs and SNPs in 14 identified ANK3 interactor proteins. ANK3 is an adaptor protein that, through interactions with KCNQ2/KCNQ3 channel complexes, directs channel localization in neuronal axon initial segment and contributes to stabilization of the neuronal resting potential. In the discovery sample, they found 31 significant interactions between 16 different ANK3 SNPs and two SNPs from KNQ2, which encodes a voltage-gated potassium channel. Twenty-eight of these interactions were also significant in the first replication GWAS. While none were significant in the second replication sample, the two discovery KCNQ2 SNPs showed significant interactions with five different ANK3 SNPs, supporting potential allelic heterogeneity.

Tomita et al. (2013) present their original work comparing human brain expression of 445 G-protein linked signaling system (GPLS) gene transcripts in selected brain regions in subjects with BP, major depressive disorder (MDD) and controls. Their findings suggest enhanced expression of several GPLS transcripts in BP and blunted expression in MDD, particularly in the anterior cingulate cortex. Orphan G-proteins, GPRC5B, and GPR37 were most consistently differentially-expressed, representing novel candidate susceptibility genes. GPR37 likely facilitate dopamine neurotransmission, while GPRC5B may be involved in spontaneous locomotor activity and response to new environments.

Schmunk and Gargus (2013) review the role of channelopathies in the pathogeneses of ASDs. They begin with an excellent overview of ion channel neurophysiology and then focus on summarizing known ion channel gene defects found in ASD. While many of the identified channel mutations do not neatly segregate with ASD within families, a number of susceptibility alleles have been strongly implicated in family-based studies. More recently, deep resequencing has identified potentially causal rare variants in ASD, including in CACNA1F, SCN1A, and SCN7A. The authors make a case that the collection of identified variants implicate calcium signaling in the pathogenesis and potential treatment targets in ASD.

O'Brien and Meisler (2013) provide a compelling review of the evidence for SCN8A mutations in sporadic human neuropsychiatric disease. They first describe the unique functional properties of the encoded protein, Nav1.6, relating those to the functional consequences of mutations therein, as observed in *in vivo* models and in human mutations. They describe more than 10 *de novo* human mutations in patients with epileptic encephalopathy and intellectual disability. Though functional data on these mutations is currently limited, it suggests gain-of-function mutations are associated with seizure disorders while loss-of-function predisposes to intellectual disability. With the advent of next-generation sequencing technology (NGS), identification of additional mutations will enhance our understanding of SCN8A mutations in the differential pathogenesis of epilepsy, movement disorders and cognitive impairment.

Judy and Zandi (2013) review recent evidence in genetic and expression studies supporting a role for voltage-gated potassium channels in the etiology of BP. They describe extant GWAS evidence for common genetic associations, noting the importance of large studies and meta-analyses in the detection of the modest effects conferred by common variants. The most consistent findings provided by GWAS to date have been for SNPs in ANK3 and several calcium channel genes; pathways-based analyses of GWAS data have converged on ion channel gene sets, more generally. The authors conclude that both statistical evidence and biological plausibility favor the involvement of ion channel genes in BP etiology.

Imbrici et al. (2013) review recent studies that implicate calcium, sodium and potassium channel genes in several neuropsychiatric disorders and describe promising pharmacologic strategies for targeting ion channels. Importantly, they note that several commercially-available psychiatric medications bind directly to and alter ion channel functioning which, in turn, alters extracellular concentrations of several neurotransmitters that have long been implicated in the pathophysiology of disease. The authors highlight several channel subtypes that have emerged as critical targets for drug development.

Finally, Martinez-Martinez et al. (2013) provide an overview of neuromuscular channelopathies as an exemplar of central nervous system (CNS) channelopathies more generally. They review the evidence supporting a role for channel and receptor autoantibodies in neuropsychiatric disease. They also describe known ion channel autoantibody targets in the brain and the diseases related to them. While most of these conditions are encephalitides with some psychiatric manifestations, the authors report findings supporting a potential role of immune mechanisms in patients with exclusively neuropsychiatric disease.

Most authors aptly note that progress in neuropsychiatric disease genetics has been stalled by the complex genetic influences involved. General consensus in the field is that this complexity is marked by extensive interactions among many genetic variants within a given affected subject. That said, evidence for causal effects of rare and *de novo* mutations is also mounting with the application of next-generation sequencing (NGS) technologies. It will be interesting to see how our understanding of disease architecture may shift as we move from an era dominated by GWAS to one marked by widespread accessibility of genome-scale NGS. Whereas sequencing was formerly limited to examination of subjects with severe, syndromic or otherwise readily identifiable phenotypes, the dropping cost of NGS now permits broader application. Of course, this shift in technological capacity will require computational and analytic strategies appropriate to the task. Otherwise, we may miss out on the insights NGS has to offer us. For example, it will be important that our methods are both robust under and able to distinguish between polygenic, interacting and heterogeneous architectures (see Thornton-Wells et al., 2004). This may be particularly true in seeking to elucidate the role of ion channel genes in neuropsychiatric disease susceptibility.

Ion channel genes are one of the largest and most functionally diverse gene sets in the human genome. Their structural diversity allows exceptional fine-tuning of cellular functioning, perhaps most evident in the enormous functional diversity of neurons. While channel genes are characterized by strong evolutionary conservation, thousands of variants have been identified as causative mutations in human disease (e.g., cystic fibrosis, multiple forms of epilepsy, familial migraine, spinocerebellar ataxias, to name but a few). These known channelopathies likely represent low-hanging fruit as most are especially severe with readily characterizable phenotypes. That said, GWAS and NGS studies in neuropsychiatric disorders are beginning to uncover channel variants that may confer disease susceptibility. Given their role in mediating highly-specialized neuronal functioning, and in differentiating function across diverse brain systems, this is not surprising. However, these identified variants are likely to be just the tip of the iceberg. For these more “subtle” and late-onset phenotypes, we should expect a much greater burden (and diversity) of mutations to be at play. While the more circumscribed set of mutations mediating severe syndromic and early-onset diseases are more amenable to detection by genetic approaches, the larger mutational target mediating risk for neuropsychiatric phenotypes will require methods that are not limited by architectural assumptions or by parametric methods. I suspect there are a great many channelopathies yet to be discovered.

## Conflict of interest statement

The author declares that the research was conducted in the absence of any commercial or financial relationships that could be construed as a potential conflict of interest.
